# Genetic Variation in NFKB1 and NFKBIA and Susceptibility to Coronary Artery Disease in a Chinese Uygur Population

**DOI:** 10.1371/journal.pone.0129144

**Published:** 2015-06-15

**Authors:** Hong-Mei Lai, Xiao-Mei Li, Yi-Ning Yang, Yi-Tong Ma, Rui Xu, Shuo Pan, Hui Zhai, Fen Liu, Bang-Dang Chen, Qian Zhao

**Affiliations:** 1 Department of Cardiology, First Affiliated Hospital of Xinjiang Medical University, Urumqi, China; 2 Department of Cardiology, People’s Hospital of Xinjiang Uygur Autonomous Region, Urumqi, China; 3 Xinjiang Key Laboratory of Cardiovascular Disease Research, Urumqi, China; 4 Clinical Research Institute of Xinjiang Medical University, Urumqi, China; 5 1st Department of Cardiology, People’s Hospital of Shaanxi Province, Xian, China; University of Milan, ITALY

## Abstract

**Objectives:**

Coronary artery disease (CAD) is the most common chronic inflammatory disease worldwide. NF-*κ*B, a central regulator of inflammation, is involved in various inflammatory diseases. The aim of this study was to investigate the association between NFKB1 and NFKBIA polymorphisms and the susceptibility to CAD and their impact on plasma levels of IL-6 in a Chinese Uygur population.

**Methods:**

We genotyped NFKB1-94ins/del ATTG (rs28362491) and NFKBIA3’ UTR A/G (rs696) using TaqMan SNP genotyping assays in 960 Uygur CAD cases and Uygur 1060 CAD-negtive controls. IL-6 plasma levels were measured in 360 stable angina pectoris (SAP) cases and 360 controls using ELISA method.

**Results:**

There was no significant difference in the distribution of the genotypes and alleles of rs696 polymorphism in CAD cases and controls. Significant difference in the frequency of genotypes (*P* = 0.001) and alleles (*P =* 0.001) of rs28362491 polymorphism was observed in CAD cases compared to controls. In multivariate logistic regression analysis, SNP rs28362491 was consistently associated with CAD risk in a recessive model after adjustment for cardiovascular risk factors (OR = 1.581, 95% CI 1.222 to 2.046, *P*<0.001). SAP cases had significantly higher plasma levels of IL-6 compared to controls (*P*<0.001). General linear model analysis showed rs28362491 was independently associated with increased IL-6 levels by analyses of a recessive model (*P*<0.001) after adjustment for covariates.

**Conclusions:**

Our study indicates that NFKB1-94 ins/del ATTG polymorphism may play a role in CAD susceptibility in Chinese Uygur population and is functionally associated with IL-6 expression, suggesting a mechanistic link between NFKB1-94 ins/del ATTG polymorphism and CAD susceptibility.

## Introduction

Coronary artery disease (CAD) is the most common chronic disease in adults, and it remains the major cause of morbidity and mortality worldwide. CAD is a complex disease caused by the interaction of environmental, genetic and inflammatory factors. The role of genetic factors in modulating susceptibility to CAD has been known for many years [1–4].

The role of inflammation in the pathogenesis of atherosclerosis is well known [5, 6]. It has received substantial attention in the past few years with numerous studies assessing its association with CAD risk. The nuclear factor *κ*B (NF-*κ*B) is a family of transcription factors that play a pivotal role in regulating inflammation, innate immune system, proliferation and apoptosis. It also regulates the expression of many genes implicated in the pathogenesis of atherosclerosis, such as cytokines, chemokines, adhesion molecules, and acute phase proteins [7–10].

NF-*κ*B family consists of 5 members: p65 (RelA), c-Rel, RelB, NF-*κ*B1 (p50/p105), and NF-*κ*B2 (p52/ p100). The major form of NF-*κ*B is a heterodimer of the p50 and p65 subunits. NFKB1 encodes both the p50 and p105 subunits of NF-*κ*B. The p50 subunit has antiinflammatory properties in its homodimer (p50/p50) form by inhibiting transcription of proinflammatory cytokines, while its heterodimer p65/p50 form has proinflammatory properties. The functional polymorphism in the promoter region of NFKB1 gene is a four base ATTG insertion/deletion variant (-94ins/del ATTG, rs28362491), encodes three differential genotypes: wild-type homozygous insertion (ins/ins), variant homozygous deletion (del/del) and heterozygous (ins/del). The NFKB1-94ins/del ATTG polymorphism produces lower protein levels of p50, having been reported to correlate with many inflammatory diseases such as Grave’s disease, ulcerative colitis, and systemic lupus erythematosus [11–13].

In the resting state, the activity of NF- *κ*B is controlled by the I*κ*B family, which binds to NF- *κ*B and sequesters them in the cytoplasm. I*κ*Bα, encoded by the NFKBIA, is the most important inhibitor of NF-*κ*B. In response to inflammatory stimuli such as LPS, reactive oxygen species (ROS), and tumor necrosis factor alpha (TNF-α), I*κ*Bα is ubiquitinated and degraded, NF-*κ*B then translocate into the nucleus, and initiate the transcription of inflammation-related genes, such as various cytokines and chemokines, thereby contributing to the pathogenesis of chronic inflammatory diseases. NFKBIA comprises several polymorphisms including -881 A/G (rs 3138053), -826 C/T (rs 2233406), -550 A/T (rs 2233407), -519 C/T (rs 2233408), and -297 C/T (rs 2233409) and 3’UTR A/G (rs 696) polymorphisms. Previous studies showed genetic polymorphism of rs696 could alter the function and structure of I*κ*Bα, thus affect the expression and activation of NF-*κ*B [14, 15].

Interleukin-6 (IL-6) is a proinflammatory cytokine with pleiotropic properties, implicated in inflammation cascade, acute phase reactions and immune responses. Its mRNA levels in atherosclerotic arteries are 10 to 40 times higher than that in nonatherosclerotic vessels [16]. IL-6 has been shown to be a key mediator of the inflammatory process, independently associated with the progression of atherosclerotic plaque and plaque instability [17, 18]. The IL-6 production is influenced by many factors including antigenic challenge, inflammation and genetic factors. In light of the pivotal role played by NFKB1 and NFKBIA in regulating many cytokines including IL-6, we hypothesized that these variants in NFKB1 and NFKBIA would have functional influence on plasma levels of IL-6 and its association with CAD risk.

Earlier analyses have shown considerable heterogeneity in genetic effects on CAD risk between Uygur and Han population in China [19–22], probably owing to the distinct ethnic origin and genetic background that are specific to Uygur population. Considering the important role played by NF-*κ*B in regulating inflammation, this raises the hypothesis that polymorphisms in NFKB1 and NFKBIA may affect CAD risk in Chinese Uygur population.

Therefore, the aim of this study was to investigate the potential association of NFKB1 and NFKBIA polymorphisms with the susceptibility to CAD and its impact on the plasma levels of IL-6 in a Chinese Uygur population.

## Materials and Methods

### Ethical approval of the study protocol

This study protocol was approved by the Ethics Committee of the First Affiliated Hospital of Xinjiang Medical University. All participants provided written informed consent to participate in genetic research.

### Study design and population

This study was a case-control association study conducted at the First Affiliated Hospital of Xinjiang Medical University. A total of 960 Uygur patients diagnosed with CAD at the First Affiliated Hospital of Xinjiang Medical University between January 2006 and December 2013 were recruited. CAD was defined as the presence of at least one significant coronary artery stenosis of more than 50% luminal diameter on coronary angiography. The CAD population comprised subgroups of cases with stable angina pectoris (SAP, n = 680) and acute coronary syndrome (ACS, n = 280). Exclusion criteria were those who have incomplete inhospital data collection; those diagnosed with valvular heart disease, congenital heart disease, and non-ischaemic cardiomyopathy.

We randomly sampled 1060 ethnic and geographic matched individuals for the control group. All control subjects were from the Cardiovascular Risk Survey (CRS) study. The design of CRS study has been previously reported [23, 24]. In brief, it is a cross-sectional study of risk factors for cardiovascular diseases in the multiethnic population (mainly Han, Uygur, Hazakh population) in Xinjiang which was performed from June 2007 to March 2010. The study consists of interviews, physical examinations, and data from blood sample analyses. Individuals were excluded from this study if they have: a history of CAD; electrocardiographic signs of CAD; regional wall motion abnormalities; relevant valvular abnormalities in echocardiograms and/or carotid atherogenesis [25].

### Definition of cardiovascular risk factors

Hypertension was defined as history of hypertension and/or an average systolic blood pressure ≥ 140 mmHg and/or an average diastolic blood pressure ≥ 90 mmHg. Participants were considered diabetic if they reported a physician diagnosis of diabetes or were taking anti-diabetic medication or had fasting/non-fasting glucose ≥ 126 mg/dL/ ≥ 200 mg/dL. Body mass index (BMI) was calculated by dividing the weight in kilograms by the height in meters squared. Persons reporting regular tobacco use in the previous 6 months were considered as current smokers. SAP was defined as no change in frequency, duration, or intensity of angina in the 6 weeks before percutaneous coronary intervention. ACS was defined as worsening of angina pectoris, and/or acute myocardial infarction.

### Routine blood test

Fasting peripheral blood samples were obtained from all participants for the assessment of routine biochemical variables. Total cholesterol (TC), Low density lipoprotein (LDL) and High density lipoprotein-cholesterol (HDL), and Triglycerides (TG) were measured using standard enzymatic methods in the Central Laboratory of the First Affiliated Hospital of Xinjiang Medical University.

### DNA isolation and Single-nucleotide polymorphism genotyping

Genomic DNA was extracted from peripheral vein blood leukocytes using a whole blood genome extraction kit (TIANGEN Bioteck coperation, Beijing, China) following the manufacturer’s instructions. The DNA samples were shipped to the core laboratory for storage at −80°C until analysis. The concentration and purity of DNA samples were established using a NanoDrop spectrophotometer. DNA samples with concentration 50 ng/μL were included in the study.

All participants were typed for NFKB1 and NFKBIA gene polymorphisms (SNP rs 28362491, SNP rs696). Genotyping was carried out using the TaqMan SNP Genotyping Assay (Applied Biosystems). The primers and probes used in the TaqMan SNP Genotyping Assays (ABI) were chosen based on information at the ABI website (http://myscience.appliedbiosystems.com). The endpoint was read after PCR amplification using an Applied Biosystems 7900HT Sequence Detection system. Genotyping quality was tested by including 6 blinded duplicate samples in each 96-well assay. The average agreement rate was>99%.

### Randomization and measurement of IL-6

To obtain further insights into the potential functional influence of NFKB1 and NFKBIA gene polymorphisms, we measured plasma IL-6. Considering the impact of acute coronary syndrome on plasma concentrations of IL-6, we measured plasma concentrations of IL-6 only in SAP cases and controls. 360 SAP cases and 360 controls were randomly assigned to measure the plasma concentrations of IL-6. The randomization sequence was computer generated at the Key Laboratory of Cardiovascular Disease Research of Xinjiang Medical University (blocked randomization) and participants’ allocations were kept in sequentially numbered sealed envelopes. Group allocations were issued by the secretarial staff of the Key Laboratory. IL-6 measurement was performed after successful genotyping of NFKB1 and NFKBIA polymorphisms.

Plasma concentrations of IL-6 were measured from stored frozen plasma samples using a commercial ELISA kit (R&D System, Inc., Minneapolis, MN) according to the manufacturer’s instructions.

### Statistical analysis

Continuous variables were presented as mean ± standard deviation and compared using the Student t test between groups. Categorical variables were presented as proportions and compared with the chi-square test. Chi-square tests were used to assess whether genotypes were in Hardy-Weinberg equilibrium (HWE) and to compare genotype and allele frequencies between CAD cases and control subjects. The odds ratios (ORs) of CAD and 95% confidence intervals (CIs) were calculated to assess the risk associated with each SNP and major risk factors by means of non-conditional logistic regression models in which CAD was considered as the dependent variable and the SNP genotype as the independent variables according to a recessive model. IL-6 results were calculated using an analysis of One-way ANOVA test. General linear model (GLM) analysis was performed to test for associations between SNP genotypes and serum IL-6 after adjustment for confounding variables.

A 2-sided *P*<0.05 was considered to indicate statistical significance. All statistical analyses were performed with the SPSS for Windows (version 17.0, SPSS Inc., Chicago, IL, USA).

## Results

Overall, a total of 960 CAD cases (mean age 56.99 ± 9.15 and 62.5% men) and 1060 CAD-negtive controls (mean age 56.64 ± 8.37 and 61.3% men) were included in the present study. Among them, IL-6 was randomly measured in 360 SAP cases and 360 controls.

### Characteristics of the study population

Clinical characteristics of all participants at baseline are summarized in Table 1 according to CAD status. For total participants, males, and females, CAD cases had lower HDL and higher BMI, fasting blood glucose, LDL-C, TC, and higher prevalence of hypertension, diabetes and smoking compared with controls (*P* = 0.001 for fasting blood glucose, and *P*≤0.001 for all others). There were no differences between the groups with respect to age, and TG.

**Table 1:** Basic clinical characteristics of study population according to CAD status.

	Total			Males			Females		
	CAD	Controls	*P* value	CAD	Controls	*P* value	CAD	Controls	*P* value
Number (n)	960	1060		600	650		360	410	
Age (years)	56.99±9.15	56.64±8.37	0.379	55.67±9.41	55.44±9.00	0.664	59.18±8.27	58.54±6.85	0.247
BMI (kg/m2)	27.09±2.43	26.09±2.54	<0.001*	27.24±2.50	26.70±2.37	<0.001*	26.83±2.30	25.12±2.49	<0.001*
TG (mmol/L)	1.57±0.54	1.53±0.62	0.154	1.56±0.53	1.54±0.63	0.579	1.58±0.55	1.52±0.61	0.109
TC (mmol/L)	4.41±0.66	4.19±0.59	<0.001*	4.39±0.66	4.18±0.58	<0.001*	4.45±0.65	4.22±0.60	<0.001*
HDL (mmol/L)	1.03±0.25	1.07±0.19	<0.001*	1.01±0.23	1.04±0.18	0.035*	1.05±0.28	1.12±0.19	<0.001*
LDL (mmol/L)	2.58±0.51	2.45±0.47	<0.001*	2.58±0.53	2.46±0.46	<0.001*	2.60±0.48	2.43±0.50	<0.001*
Fasting Glu (mmol/L)	5.34±1.56	5.11±1.45	0.001*	5.30±1.51	5.09±1.38	0.009*	5.42±1.64	5.15±1.57	0.020*
EH (%)	470(49.0%)	380(35.8%)	<0.001*	275(45.8%)	240(36.9%)	0.002*	195(54.2%)	140(34.1%)	<0.001*
DM (%)	213(22.2%)	143(13.5%)	<0.001*	125(20.8%)	85(13.1%)	<0.001*	88(24.4%)	58(14.1%)	<0.001*
Current smokers (%)	278(29.0%)	234(22.1)%	<0.001*	278(46.3%)	234(36.0%)	<0.001*	0	0	

BMI, body mass index; TG, triglycerides; TC, total cholesterol; HDL, high density lipoprotein; LDL, low density lipoprotein; Glu, glucose; EH, essential hypertension; DM, diabetes mellitus.

Continuous variables were expressed as mean ± standard deviation.

*P* value of continuous variables was calculated by independent *t* test.

The *P* value of categorical variable was calculated using Chi-square test.

*P<0.05

### Allele and genotype frequencies

The genotype frequencies of the two SNPs in CAD cases and controls confirmed to the Hardy–Weinberg equilibrium. The NFKB1-94ins/del ATGG and NFKBIA3’ UTR A/G genotypes and alleles frequencies in CAD cases and controls are shown in Table 2. There was no significant difference in the distribution of genotypes (AA, AG or GG), and alleles (A, G) of NFKBIA3’UTR A/G polymorphism between CAD cases and controls between sexes (for total participants, males, and females, all *P*>0.05). For total participants, males, and females, the distribution of alleles (I, D) and the recessive model (DD vs. II+ID) of NFKB1-94ins/del ATGG polymorphism differed significantly between CAD cases and controls (for allele: *P* = 0.001, *P* = 0.007, and *P* = 0.047, respectively; for recessive model: *P*<0.001, *P* = 0.007, and *P* = 0.007, respectively).

**Table 2:** Genotype and Allele Distributions in Patients with Coronary Artery Disease and Control Subjects (Uygur Population).

	Total			Males			Females		
Variants	CAD(n = 960)	Controls(n = 1060)	*P* value	CAD(n = 600)	Controls(n = 650)	*P* value	CAD(n = 360)	Controls(n = 410)	*P* value
rs696									
Genotyping									
GG	427(44.5%)	489(46.1%)	0.447	274(45.7%)	298(45.8%)	0.624	153(42.5%)	191(46.6%)	0.475
AG	418(43.5%)	462(43.6%)		262(43.7%)	293(45.1%)		156(43.3%)	169(41.2%)	
AA	115 (12.0%)	109(10.3%)		64(10.7%)	59(9.1%)		51(14.2%)	50(12.2%)	
Dominant model									
GG	427(44.5%)	489(46.1%)	0.474	274(45.7%)	298(45.8%)	0.955	153(42.5%)	191(46.6%)	0.276
AG+AA	533(45.5%)	571(53.9%)		326(54.3%)	352(54.2%)		207(57.5%)	219(53.4%)
Additive model									
AG	418(43.5%)	462(43.6%)	0.984	262(43.7%)	293(45.1%)	0.649	156(43.3%)	169(41.2%)	0.559
GG+AA	542(56.5%)	598(56.4%)		338(56.3%)	357(54.9%)		204(56.7%)	241(58.8%)	
Recessive model									
AA	115 (12.0%)	109(10.3%)	0.229	64(10.7%)	59(9.1%)	0.392	51(14.2%)	50(12.2%)	0.454
GG+AG	845(88.0%)	951(89.7%)		536(89.3%)	591(90.9%)		309(85.8%)	360(87.8%)	
Allele									
G	1272(66.3%)	1440(67.9%)	0.268	810(67.5%)	889(68.4%)	0.637	462(64.2%)	551(67.2%)	0.216
A	648(33.7%)	680(32.1%)		390(32.5%)	411(31.6%)		258(35.8%)	269(32.8%)	
rs28362491									
Genotyping									
II	357(37.2%)	437(41.2%)	0.001*	229(38.2%)	280(43.0%)	0.017*	128(35.6%)	157(37.3%)	
ID	425(44.3%)	492(46.4%)		263(43.8%)	289(44.5%)		162(45.0%)	203(49.5%)	0.022*
DD	178(18.5%)	131(12.4%)		108(18.0%)	81(12.5%)		70(19.4%)	50(12.2%)	
Dominant model									
II	357(37.2%)	437(41.2%)	0.068	229(38.2%)	280(43.0%)	0.084	128(35.6%)	157(37.3%)	
ID+DD	603(63.8%)	623(58.8%)		371(61.8%)	370(57.0%)		232(64.4%)	253(62.7%)	0.455
Additive model									
ID	425(44.3%)	492(46.4%)	0.347	263(43.8%)	289(44.5%)	0.864	162(45.0%)	203(49.5%)	
II+DD	535(55.7%)	568(53.6%)		337(56.2%)	361(55.5%)		198(55.0%)	207(50.5%)	0.219
Recessive model								
DD	178(18.5%)	131(12.4%)	<0.001*	108(18.0%)	81(12.5%)	0.007*	70(19.4%)	50(12.2%)	
II+ID	782(81.5%)	929(87.6%)		492(82.0%)	569(87.5%)		290(80.6%)	360(87.8%)	0.007*
Allele								
I	1139(59.3%)	1366(64.4%)	0.001*	721(60.1%)	849(65.3)	0.007*	418(58.1%)	517(63.0%)	
D	781(40.7%)	754(35.6%)		479(39.9%)	451(34.7%)		302(41.9%)	303(37.0%)	0.047*

The *P*-value of genotype was calculated by Chi-square test.

*P<0.05.

### Association of the NFKB1-94ins/del ATTG and NFKBIA3’ UTR A/G polymophisms with CAD risk

Univariate and multivariate logistic regression analysis of the association between CAD and multiple parameters stratified by gender are presented in Table 3. In a multivariate logistic regression model with CAD as the dependent variable, we identified a significant association of rs28362491 with CAD for males and females in a recessive model (for total, OR = 1.581, 95% CI 1.222 to 2.046, *P*<0.001). In the male group, individuals with NFKB1 del/del genotype had 1.535-fold increased risk of developing CAD than individuals with ins/ins+ins/del genotypes (*P* = 0.010). In the female group, NFKB1 del/del genotype was also an independent risk factor of CAD (OR = 1.599, 95% CI 1.025 to 2.495, *P* = 0.039) after adjustment for kown cardiovascular risk factors, indicating that the effect of NFKB1-94ins/del ATTG polymorphism was not mediated by an effect on other cardiovascular risk factors. No significant evidence of heterogeneity was observed in the effect of the NFKBIA 3’UTR A/G polymorphism on CAD risk between CAD cases and controls for males, and females (All *P*>0.05).

**Table 3:** Multiple logistic regression analysis for Uygur CAD patients and control subjects.

	Total			Males			Females		
	OR	95%CI	*P*	OR	95%CI	*P*	OR	95%CI	*P*
rs28362491	1.581	1.222–2.046	<0.001*	1.535	1.110–2.122	0.010*	1.599	1.025–2.495	0.039*
(DD vs II + ID)									
Smoking	1.301	1.050–1.611	0.016*	1.485	1.172–1.882	0.001*			
BMI	1.149	1.106–1.194	<0.001*	1.070	1.020–1.123	0.006*	1.355	1.259–1.457	<0.001*
EH	1.596	1.322–1.926	<0.001*	1.357	1.071–1.720	0.011*	2.051	1.485–2.833	<0.001*
DM	1.947	1.285–2.948	0.002*	1.815	1.111–2.964	0.017*	2.422	1.096–5.355	0.029*
Glu	0.930	0.839–1.031	0.167	0.959	0.846–1.087	0.514	0.860	0.711–1.039	0.118
TG	0.964	0.821–1.132	0.652	0.924	0.756–1.128	0.436	0.988	0.747–1.307	0.934
TC	1.587	1.361–1.850	<0.001*	1.567	1.294–1.898	<0.001*	1.653	1.264–2.162	<0.001*
HDL	0.520	0.335–0.806	0.003*	0.734	0.409–1.315	0.298	0.268	0.130–0.550	<0.001*
LDL	1.485	1.221–1.806	<0.001*	1.485	1.163–1.897	0.002*	1.615	1.151–2.267	0.006*

EH, essential hypertension; DM, diabetes mellitus; CAD, coronary artery disease.

### Subgroup analysis of the NFKB1-94ins/del ATTG and NFKBIA3’ UTR A/G polymorphisms

We further stratified our overall group of CAD cases based on the different categories of CAD. In SAP subgroup, we found NFKB1 del/del genotype was significantly associated with increased risk of SAP for males, and females (for males, OR = 1.538, 95%CI 1.077 to 2.194, *P* = 0.018; for females, OR = 1.650, 95%CI 1.031 to 2.639, *P* = 0.037, respectively) (Table 4). In ACS subgroup, we observed the risk of developing ACS was different between men and women. In the male group, individuals with NFKB1 del/del genotype had a 1.620-fold increased risk of developing ACS. No female-specific association of NFKB1-94ins/del ATTG polymorphism was observed (Table 5). For males, and females, no significant evidence of heterogeneity was observed in the effect of the NFKBIA3’UTR A/G polymorphism on SAP and ACS risk between CAD cases and controls (All *P*>0.05) (Tables 4 and 5).

**Table 4:** Subgroup analysis of studied polymorphisms with risk of SAP.

Genotypes	Total				Males				Females			
	Controls	SAP	OR*(95%CI)	*P*	Controls	SAP	OR*(95%CI)	*P*	Controls	SAP	OR*(95%CI)	*P*
	n = 1060	n = 680			n = 650	n = 420			n = 410	n = 260		
Rs28362491												
II	437(41.2%)	254(37.4%)	0.822(0.668–1.012)	0.064	280(43.0%)	162(38.6%)	0.843(0.651–1.093)	0.198	157(38.3%)	92(35.4%)	0.813(0.568–1.164)	0.258
ID	492(46.4%)	301(44.3%)	0.944(0.771–1.156)	0.576	289(44.5%)	184(43.8%)	0.948(0.733–1.224)	0.681	203(49.5%)	117(45.0%)	0.927(0.657–1.309)	0.667
DD	131(12.4%)	125(18.4%)	1.599(1.121–2.113)	0.001*	81(12.5%)	74(17.6%)	1.538(1.077–2.194)	0.018*	50(12.2%)	51(19.6%)	1.650(1.031–2.639)	0.037*
Rs696												
GG	489(46.1%)	293(43.1%)	0.890(0.726–1.090)	0.260	298(45.8%)	184(43.8%)	0.910(0.704–1.175)	0.468	191(46.6%)	109(41.9%)	0.902(0.637–1.278)	0.563
AG	462(43.6%)	306(45.0%)	1.045(0.852–1.280)	0.675	293(45.1%)	190(45.2%)	0.997(0.772–1.287)	0.981	169(41.2%)	116(44.6%)	1.114(0.786–1.580)	0.544
AA	109(10.3%)	81(11.9%)	1.203(0.873–1.657)	0.259	59(9.1%)	46(11.0%)	1.313(0.860–2.005)	0.208	50(12.2%)	35(13.5%)	0.990(0.594–1.649)	0.969

OR*: adjusted for smoking, hypertension, diabetes, BMI, TC, HDL-C, and LDL.

**Table 5:** Subgroup analysis of studied polymorphisms with risk of ACS.

Genotypes	Total				Males				Females			
	Controls	ACS	OR*(95%CI)	*P*	Controls	ACS	OR*(95%CI)	*P*	Controls	ACS	OR*(95%CI)	*P*
	n = 1060	n = 280			n = 650	n = 180			n = 410	n = 100		
Rs28362491												
II	437(41.2%)	103(36.8%)	0.840(0.630–1.119)	0.233	280(43.0%)	67(37.2%)	0.803(0.565–1.143)	0.223	157(38.3%)	36(36.0%)	0.968(0.578–1.621)	0.902
ID	492(46.4%)	124(44.3%)	0.938(0.709–1.241)	0.655	289(44.5%)	79(43.9%)	0.955(0.676–1.350)	0.794	203(49.5%)	45(45.0%)	0865(0.525–1.425)	0.568
DD	131(12.4%)	53(18.9%)	1.548(1.065–2.248)	0.022*	81(12.5%)	34(18.9%)	1.620(1.026–2.557)	0.038*	50(12.2%)	19(19.0%)	1.419(0.712–2.828)	0.320
Rs696												
GG	489(46.1%)	134(47.9%)	1.062(0.804–1.403)	0.672	298(45.8%)	90(50.0%)	1.143(0.813–1.608)	0.441	191(46.6%)	44(44.0%)	0.978(0.591–1.619)	0.932
AG	462(43.6%)	112(40.0%)	0.853(0.643–1.132)	0.270	293(45.1%)	72(40.0%)	0.818(0.578–1.157)	0.257	169(41.2%)	40(40.0%)	0.879(0.527–1.467)	0.622
AA	109(10.3%)	34(12.1%)	1.272(0.825–1.963)	0.277	59(9.1%)	18(10.0%)	1.204(0.674–2.152)	0.531	50(12.2%)	16(16.0%)	1.342(0.668–2.697)	0.409

OR*: adjusted for smoking, hypertension, diabetes, BMI, Glu, TG, TC, HDL-C, and LDL.

### NFKB1-94ins/del ATTG and NFKBIA3’UTR A/G genotypes and IL-6 levels

**Fig 1:**
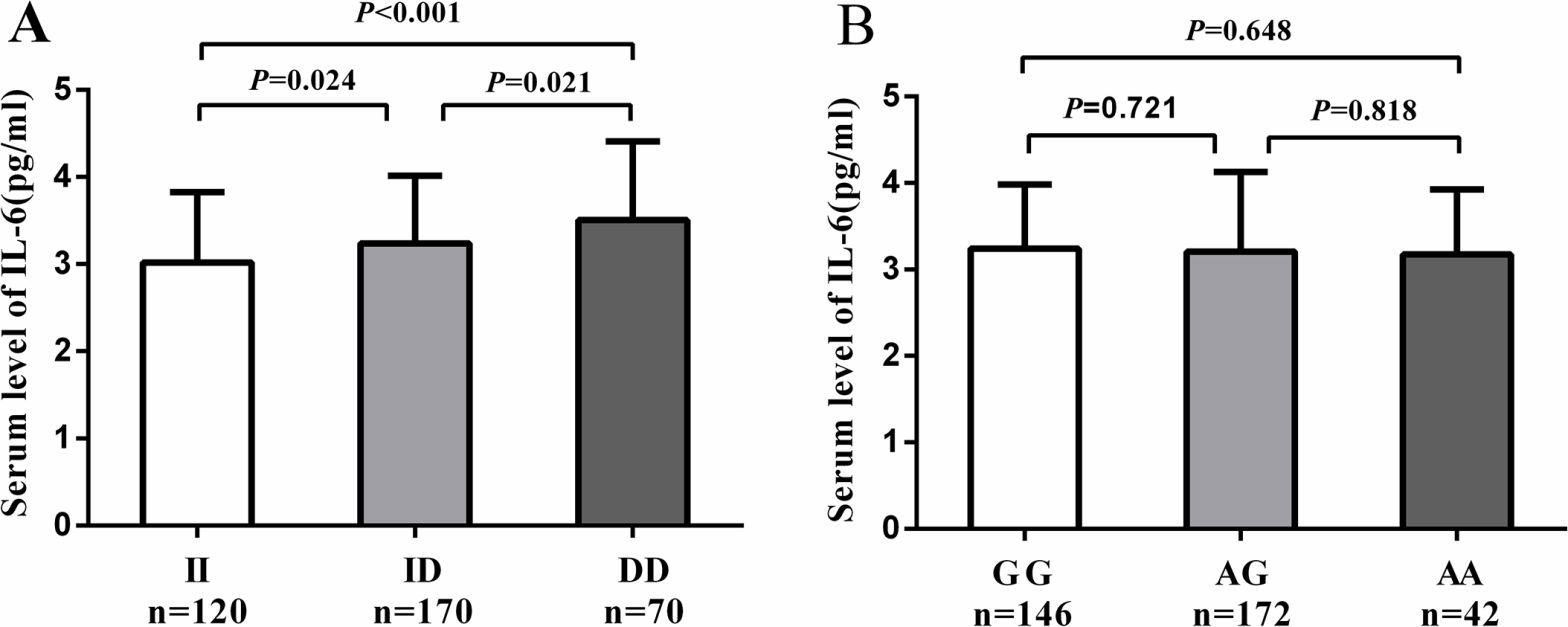
Association of IL-6 plasma levels with NFKB1-94 ins/del ATTG and NFKBIA3’UTR A/G genotypes in SAP patients. A. Mean plasma levels of IL-6 in SAP cases according to rs28362491 genotypes. DD vs ID: 3.51 ± 0.91 vs 3.24 ± 0.78, *P =* 0.021. DD vs II: 3.51 ± 0.91 vs 3.02 ± 0.81, *P<*0.001. ID vs II: 3.24 ± 0.78 vs 3.02 ± 0.81, *P =* 0.024. B. Mean plasma levels of IL-6 in SAP cases according to rs696 genotypes. AA vs AG: 3.17 ± 0.75 vs 3.21 ± 0.92, *P =* 0.818. AA vs GG: 3.17 ± 0.75 vs 3.24 ± 0.74, *P =* 0.648. AG vs GG: 3.21 ± 0.92 vs 3.24 ± 0.74, *P =* 0.721

Table 6 shows the clinical characteristics of 360 SAP cases and 360 controls included in IL-6 analysis. The mean plasma concentration of IL-6 was significantly higher in SAP cases compared to controls (3.22 ± 0.83 pg/ml vs. 2.75 ± 0.84 pg/ml, *P*<0.001). When we compared plasma IL-6 levels according to the genotypes of NFKB1-94ins/del ATTG and NFKBIA3’UTR A/G in SAP cases, the mean plasma concentration of IL-6 was significantly higher in SAP cases with del/del genotype than in cases with ins/del genotype and ins/ins genotype (3.51 ± 0.91 pg/ml vs. 3.24 ± 0.78 pg/ml, *P =* 0.021, 3.51 ± 0.91 pg/ml vs. 3.02 ± 0.81 pg/ml, *P <*0.001, respectively) (Fig 1A). The mean plasma concentration of IL-6 was comparable in SAP cases with different genotypes of NFKBIA3’UTR A/G polymorphism (AA vs. GG: 3.17 ± 0.75 pg/ml vs. 3.24 ± 0.74 pg/ml, *P =* 0.648; AA vs AG: 3.17 ± 0.75 pg/ml vs 3.21 ± 0.92 pg/ml, *P =* 0.818; AG vs. GG: 3.21 ± 0.92 pg/ml vs. 3.24 ± 0.74 pg/ml, *P =* 0.721, respectively) (Fig 1B). General linear model analysis showed that rs28362491 was associated with IL-6 levels by analyses of a dominant model (*P*<0.001) and a recessive model (*P*<0.001), and the difference remained significant after adjustment for age, sex, smoking, BMI, hypertension, diabetes, glucose, TG, TC, HDL, and LDL (Table 7). The NFKB1 del/del genotype was associated with an increased IL-6 concentration of 0.515 pg/ml (95%CI 0.313 to 0.717, *P*<0.001) compared with the ins/ins genotype, and the del/del genotype was associated with an increased IL-6 concentration of 0.331 pg/ml (95% CI 0.136–0.526, *P* = 0.001) compared with the ins/del genotype, indicating there is gene-dosage effect of the D allele on plasma levels of IL-6.

**Table 6:** Clinical features of SAP cases and healthy controls included in IL-6 analysis.

Characteristics	SAP	Controls	*P*
	n = 360	n = 360	
Age (years)	56.56±9.04	55.52±8.49	0.110
Male (%)	216(60.0%)	225(62.5%)	0.541
BMI	27.19±2.49	26.19±2.53	<0.001*
Smoking (%)	106(29.4%)	73(20.3%)	0.006*
Hypertension (%)	176(48.9%)	113(31.4%)	<0.001*
Diabetes (%)	84(23.3%)	58(16.1%)	0.019*
Fasting glucose (mmol/L)	5.37±1.64	5.19±1.50	0.112
TG (mmol/L)	1.51±0.53	1.51±0.61	0.991
TC (mmol/L)	4.42±0.66	4.22±0.59	<0.001*
HDL (mmol/L)	1.02±0.24	1.06±0.18	0.009*
LDL (mmol/L)	2.57±0.48	2.49±0.47	0.027*
IL-6 (pg/mL)	3.22±0.83	2.75±0.84	<0.001*
Genotypes			
Rs28362491			
II (%)	120(33.3%)	134(37.2%)	
ID (%)	170(47.2%)	178(49.5%)	0.080
DD (%)	70(19.5%)	48(13.3%)	
Rs696			
GG (%)	146(40.5%)	142(39.4%)	
AG (%)	172(47.8%)	178(49.5%)	0.902
AA (%)	42(11.7%)	40(11.1%)	

*P<0.05

**Table 7:** IL-6 levels and rs28362491 genotypes.

SNP	Uajusted model		Ajusted model*	
Rs28362491 genotypes	IL-6(pg/mL) Mean±SD	*P*	IL-6(pg/mL) Mean±SE	*P*
Additive model				
ID (= 348)	2.99±0.83	0.747	2.99±0.05	0.728
II+DD (n = 372)	2.97±0.90		2.97±0.05	
Dominant model				
II (n = 254)	2.81±0.83	<0.001	2.81±0.005	<0.001
ID+DD (n = 466)	3.08±0.87		3.08±0.04	
Recessive model				
DD (n = 118)	3.33±0.96	<0.001	3.32±0.08	<0.001
II+ID (n = 602)	2.92±0.83		2.92±0.04	

* Analysis of covariance adjusted for age, sex, smoking, BMI, hypertension, diabetes, glucose, TG, TC, HDL, and LDL.

## Discussion

Genetic variants play an important role in the inter-individual variation in CAD. More than 40 chromosomal loci associated with CAD in the general population have been identified. However, their cumulative effect can only explain minority of the etiology of CAD. The differences in genetic effects between Uygur and Han Chinese population raise the hypothesis that genetic predictors of CAD might exist that are specific to Uygur population.

Inflammation plays a vital role in the initiation and progression of atherosclerosis, implicating the involvement of inflammatory cytokines in the atherosclerotic processes. In the present study, we investigated the association of polymorphisms in NFKB1 and NFKBIA with the susceptibility to CAD in a Chinese Uygur population. We have identified NFKB1-94ins/del ATTG polymorphism is associated with increased CAD risk in Chinese Uygur population, and it is also associated with IL-6 levels in SAP cases, indicating the NFKB1-94ins/del ATTG polymorphism may affect CAD risk by modulating the expression of IL-6.

The role of NF-κB in inflammation is determined by its subunit type. NFKB1, the gene whose expression is influenced by the NFKB1-94ins/del ATTG polymorphism in the promoter region, encodes p50 subunit of NF-*κ*B. The p50/p50 homodimer has antiinflammatory properties by inhibiting transcription of pro-inflammatory cytokines like TNF and IL-12, and stimulating transcription of the anti-inflammatory cytokine IL10. While p65/p50 heterodimer has proinflammatory properties by stimulating the transcription of the pro-inflammatory cytokines such as TNF and IL-1β. The variant allele containing the deletion results in lower promoter transcriptional activity and lower levels of p50. P65/p50 herterodimer was less affected by the decreased p50 synthesis than p50/p50 homodimer, thus the NFKB1-94ins/del ATTG polymorphism may influence the susceptibility to inflammatory diseases by imbalancing the pro-inflammatory and anti-inflammatory response.

The NFKB1-94ins/del ATTG polymorphism has been extensively studied in cardiovascular diseases. However, so far contrasting results have been reported.Vogel et al [26] investigated the association of the NFKB1-94ins/del ATTG polymorphism with risk of coronary heart disease (CHD), demonstrating that NFKB1ins/del genotype was associated with a higher risk of CHD in Caucasians. Mishra et al [27] found that NFKB1 del/del genotype was significantly associated with left ventricular dysfunction and myocardial infarction (MI). However, Boccardi et al [28] reported NFKB1-94ins/del ATTG polymorphism was associated with lower MI susceptibility. In our study, NFKB1-94ins/del ATTG polymorphism has been analyzed in 960 CAD cases and 1060 CAD-negative controls. According to our findings, we found CAD cases had significantly higher distribution of NFKB1 del/del genotypes compared to controls. Individuals with del/del genotype had 1.581-fold increased risk for CAD as compared with individuals with ins/del and ins/ins genotype. The association between the del/del genotype and increased CAD risk was retained after adjustment of major risk factors, indicating that the NFKB1-94ins/del ATTG polymorphism may affect CAD risk through pathways beyond established risk factors. In the ACS group, the association of NFKB1-94ins/del ATTG polymorphism with ACS risk was only limited to males when the distribution of NFKB1-94ins/del ATTG genotype was analyzed separately in women and men. A possible reason for this discrepancy may be related to the small samples of ACS female cases and gender-specific cardiovascular risk factors in our substudy population.

The SNP polymorphism (rs969) in the 3’UTR region of the NFKBIA gene has been studied in some inflammatory diseases. Romozova et al [29] investigated the A/G point variation in the 3’UTR region of NFKBIA gene in Czech and German cases with type 2 diabetes, demonstrated that AA genotype of 3’UTR A/G variant is associated with diabetes risk. Koc et al [30] investigated the relationship between 3’UTR A/G polymorphism and NFKB1-94ins/del ATTG polymorphism, and the risk of Hashimoto thyroiditis in a Turkish Population, concluded that ins/ins/GG combined genotype had protective effect on the disease and this protectiveness was based on G allele of NFKBIA3’UTR A/G polymorphism. To the best of our knowledge, there have been no reports on the effects of 3’UTR A/G polymorphism of NFKBIA on CAD risk in Chinese population. In our study, no significant evidence for genetic association between any genotypes of NFKBIA3’UTR A/G and the susceptibility to CAD was observed. Our results indicate NFKBIA3’UTR A/G polymorphism is compatible with chance in Chinese Uygur population. The absence of association between the genetic variant of NFKBIA3’UTR A/G and CAD risk seems to exclude NFKBIA3’UTR A/G polymorphism as the underlying mechanism of CAD in Chinese Uygur population.

IL-6 is a pleiotropic glycoprotein cytokine secreted by the activated inflammatory cells within the vessel wall. Circulating IL-6 is increased in some autoimmune and chronic inflammatory diseases, such as diabetes, arthritis, Grave’s disease and atherosclerosis. IL-6 has several roles in the pathophysiology of atherosclerosis. It is involved in inflammatory cell recruitment and activation, induces the expression of hepatic acute-phase reactants, and stimulates the synthesis of fibrinogen. Previous studies have shown that elevated IL-6 level is associated with increased risk and severity of CAD [17, 31–33]. Kume et al [34] found antiinflammatory treatment with tocilizumab (an IL-6 receptor antagonist) is effective in reducing arterial stiffness after 24 weeks of therapy in rheumatoid arthritis patients. It is well known the ultimate consequence of NF-*κ*B signalling is the activation of inflammatory genes such as IL-6, IL-10, and IL-12 etc. The effects of NFKB1-94ins/del ATTG on plasma levels of IL-10 and IL-12 have been reported previously. However, few data exist concerning the impact of NFKB1 and NFKBIA gene polymorphisms on plasma levels of IL-6 in the context of CAD.

In our study, plasma IL-6 was measured in 360 SAP cases and 360 controls. We observed that SAP cases had significantly higher plasma IL-6 levels compared to controls. Our finding is consistent with previous studies, supporting the essential role of IL-6 in the pathogenesis of atherosclerosis. When we analyzed IL-6 according to the genotypes, we found that the NFKB1-94ins/del ATTG polymorphism is associated with IL-6 concentration. Compared with ins/ins genotype and ins/del genotype, individuals with del/del genotype had significantly higher IL-6 levels. This association remains significant after adjustment for age, sex, smoking, BMI, hypertension, diabetes, glucose, TG, TC, HDL, and LDL, indicating the association of del/del genotype with CAD risk may be in part mediated by its impact on plasma IL-6 level which may provide a mechanistic link between NFKB1 polymorphism and CAD susceptibility in Chinese Uygur population. The association of NFKB1-94ins/del ATTG polymorphism and increased plasma IL-6 concentration should be due to the reduced NFKB1 promoter activity caused by the deletion of ATTG repeat in the promoter region of NFKB1 gene and the consequent effects of increased p65/p50 heterodimer on IL-6 gene transcription.

There was no difference in plasma concentrations of IL-6 between different genotypes of NFKBIA3’ UTR A/G in SAP cases and controls, indicating that the association between IL-6 plasma levels and NFKBIA3’ UTR A/G polymorphism may be non-causal.

Our study has several limitations. First, this is a single-center experience representing a relatively small numbers of patients. Second, our study was only restricted to Chinese Uygur population, whether our findings can be extended to other races remains to be determined. Second, the plasma levels of IL-6 were measured only in a small sample size, and several other gene polymorphisms have been reported to affect plasma levels of IL-6, including IL-6 gene and IL-6 receptor polymorphisms. Further studies are needed to clarify the role of these polymorphisms on plasma level of IL-6 in CAD cases with NFKB1 del/del genotype.

In summary, our study demonstrated that the functional promoter NFKB1-94 ins/del ATTG polymorphism may play a role in CAD susceptibility in Chinese Uygur population. Our preliminary analysis indicates that the NFKB1-94ins/del ATTG polymorphism may modulate CAD risk by affecting plasma IL-6 levels. Further studies are needed to confirm our findings in different population.
